# Homologous and Heterologous Protection of Nonhuman Primates by Ebola and Sudan Virus-Like Particles

**DOI:** 10.1371/journal.pone.0118881

**Published:** 2015-03-20

**Authors:** Kelly L. Warfield, John M. Dye, Jay B. Wells, Robert C. Unfer, Frederick W. Holtsberg, Sergey Shulenin, Hong Vu, Dana L. Swenson, Sina Bavari, M. Javad Aman

**Affiliations:** 1 Integrated Biotherapeutics, Inc., Gaithersburg, Maryland, United States of America; 2 United States Army Medical Research Institute of Infectious Diseases, Fort Detrick, Maryland, United States of America; Division of Clinical Research, UNITED STATES

## Abstract

Filoviruses cause hemorrhagic fever resulting in significant morbidity and mortality in humans. Several vaccine platforms that include multiple virus-vectored approaches and virus-like particles (VLPs) have shown efficacy in nonhuman primates. Previous studies have shown protection of cynomolgus macaques against homologous infection for Ebola virus (EBOV) and Marburg virus (MARV) following a three-dose vaccine regimen of EBOV or MARV VLPs, as well as heterologous protection against Ravn Virus (RAVV) following vaccination with MARV VLPs. The objectives of the current studies were to determine the minimum number of vaccine doses required for protection (using EBOV as the test system) and then demonstrate protection against Sudan virus (SUDV) and Taï Forest virus (TAFV). Using the EBOV nonhuman primate model, we show that one or two doses of VLP vaccine can confer protection from lethal infection. VLPs containing the SUDV glycoprotein, nucleoprotein and VP40 matrix protein provide complete protection against lethal SUDV infection in macaques. Finally, we demonstrate protective efficacy mediated by EBOV, but not SUDV, VLPs against TAFV; this is the first demonstration of complete cross-filovirus protection using a single component heterologous vaccine within the *Ebolavirus* genus. Along with our previous results, this observation provides strong evidence that it will be possible to develop and administer a broad-spectrum VLP-based vaccine that will protect against multiple filoviruses by combining only three EBOV, SUDV and MARV components.

## Introduction

Ebolaviruses and marburgviruses are non-segmented, negative-strand RNA viruses belonging to the *Filoviridae* family, *Mononegavirales* order. The genus *Ebolavirus* has five members: Ebola virus (EBOV), Sudan virus (SUDV), Taï Forest virus (TAFV), Reston virus (RESTV) and Bundibugyo virus (BDBV) [1]. The *Marburgvirus* genus has two members, Marburg virus (MARV) and Ravn virus (RAVV) [2]. Filoviruses cause a hemorrhagic fever disease that is highly lethal with case fatality rates of 30–90% during outbreaks in humans caused by EBOV, SUDV, BDBV, RAVV, and MARV [3]. In contrast, RESTV has not caused any known disease in humans, [4] and only a single non-lethal case has been reported for TAFV [5].

The filovirus genome consists of seven genes encoding seven major proteins in the case of MARV and RAVV, and nine major proteins in the case of ebolaviruses. The viral proteins (VP)30, VP35, and nucleoprotein (NP) encapsidate the negative-stranded genome to form the nucleocapsid structure. VP40 is the major matrix protein and the main protein that triggers budding of filamentous particles; VP24 is considered a minor matrix protein. The trimeric glycoprotein (GP) is expressed on the surface and contains the receptor binding region and the ectodomain required for fusion. GP appears to be the primary determinant for protection against lethal infection, although other proteins can also play a role [6]. GP and VP40 can assemble into virus-like particles (VLPs) when expressed ectopically in mammalian or insect cells [7–10], and other viral proteins such as NP and VP24 can also be incorporated into the particles [7, 9–12].

VLPs represent a promising vaccine platform for a diverse array of viruses that include: influenza virus, rotaviruses, noroviruses, HIV, hepatitis B virus, parvoviruses, rift valley fever virus, human papillomavirus and also filoviruses [13–17]. A significant advantage of VLPs is their similar morphology to their replication competent ‘parent’ viruses, thus allowing protective antigens to be presented to the immune system in a similar manner to the infectious human pathogen [18–20]. Likely due to their authentic structures, VLPs can stimulate powerful innate, humoral and cellular immune responses [13, 14]. VLP-based vaccines appear to represent a safe and effective prophylactic countermeasure for filovirus hemorrhagic fever. The filovirus vaccine candidate tested most extensively to date is an enveloped VLP with the glycoprotein on the surface inserted into the lipid bilayer, a layer of VP40 underneath the membrane, and NP (when included), localized in the core beneath VP40. The VLPs have variable morphology ranging from nearly spherical to long, filamentous structures with a diameter of approximately 70–100 nm and length of 400–600 nm [7–10].

Vaccination of cynomolgus macaques with EBOV or MARV VLPs elicits rapid and robust humoral and cell-mediated immune responses leading to protection against infection with lethal homologous virus [21, 22]. We have previously shown that EBOV VLPs containing the EBOV GP, NP, and VP40 proteins, generated in mammalian cells and administrated at a dose of 250 μg via intramuscular injection 3 times at 42 day intervals, induce humoral and cellular responses in mice [23] and NHPs [21]. Following a normally lethal EBOV challenge, the EBOV VLP-vaccinated macaques were protected with no overt signs of clinical disease such as rash, anorexia, or weight loss. Moreover, there was no detectable viremia in any of the vaccinated animals [21]. More recent vaccine studies in rodents were performed with VLPs produced in either Hi5 or Sf9 insect cells using a recombinant baculovirus expression system [11, 22]. Baculovirus/insect cell-derived EBOV VLPs induce dendritic cell activation, neutralizing antibody and cellular immune responses that provide protection in rodents [11, 24, 25]; however, protection in nonhuman primates has not been previously documented. Furthermore, efficacy of VLP based vaccines against SUDV has not previously been reported. MARV VLPs produced using the recombinant baculovirus/insect cell system protect NHPs against multiple marburgviruses including MARV (MARV-Musoke and -Ci67 isolates) and RAVV [22]. These data suggest that cross variant/filovirus protection may be possible for MARV. Cross species partial protection for ebolaviruses has been shown only for an EBOV based VSV vectored vaccine against BDBV infection [26].

The goal of the current study was to investigate in nonhuman primates if: *i)* two vaccinations with EBOV VLPs would be sufficient for protection against EBOV infection; *ii)* VLPs based on SUDV provide protection against homologous infection; and *iii)* whether cross species protection can be achieved between EBOV, SUDV, and a third ebolavirus, TAFV.

## Materials and Methods

### Generation of Ebola and Marburg VLPs

For expression in an insect cell system, homologous GP, NP, and VP40 genes were cloned into a transfer vector containing the polyhedrin late promoter and SV40 polyadenylation site. Bacmid DNA was generated by *in vivo* transposition in *E*. *coli*, and DNA was transfected into Sf9 insect cells. Recombinant baculoviruses containing the filovirus proteins of interest were recovered from supernatants and amplified with another passage through Sf9 cells. The final viruses were introduced into Hi5 or Sf9 insect cells, the VLPs were recovered from the culture supernatants by high-speed concentration and subsequent purification on sucrose gradients, as published elsewhere [7–9, 27]. Before use in animals, VLP vaccine preparations were characterized using a battery of assays including total protein (BCA), identity (Western blotting using mouse monoclonal or epitope-specific rabbit antibodies recognizing EBOV or SUDV GP, VP40, and NP), electron microscopy, and endotoxin content, as previously described [7–9, 27]. Mouse monoclonal antibodies were obtained from USAMRIID or produced internally using established hybridoma cell lines. Rabbit antibodies were produced by vaccination with peptides and purified IgG fractions were obtained (Genscript, Piscataway, NJ).

### Filoviruses

Live EBOV-Kikwit, SUDV-Boniface and TAFV were propagated and enumerated by a standard plaque assay on Vero or Vero E6 cells using a solid agarose overlay for 6–7 days followed by staining with neutral red for visualization of the plaques [28]. Filovirus-infected cells and animals were handled by qualified personnel under maximum containment in a biosafety level-4 laboratory at the United States Army Medical Research Institute of Infectious Diseases.

### Nonhuman primate studies

Research was conducted in compliance with the Animal Welfare Act and other federal statutes and regulations relating to animals and experiments involving animals and adhered to principles stated in the *Guide for the Care and Use of Laboratory Animals*, National Research Council, 1996. The facilities where the research was conducted are fully accredited by the Association for Assessment and Accreditation of Laboratory Animal Care International. Vaccination portions of the studies were conducted under BSL-2 conditions at Covance (Denver, PA) and USAMRIID (Fort Detrick, MD) with all live virus challenges occurring under BSL-4 containment conditions at USAMRIID. All studies were approved by the relevant Institutional Animal Care and Use Committee at Covance or USAMRIID. All animal housing areas at both sites are continuously monitored for temperature and humidity using a state of art monitoring system, results are assessed regularly and recorded to ensure animal health and welfare. Animals were individually housed in stainless cages and provided environmental enrichment such as mirrors and toys at both sites and music (Covance only). Animals were supplied with primate diet (ex. Purina 5L07 diet) throughout the study. In addition, animals were also provided with treats such as fresh fruits and vegetables. Water was available *ad libitum* throughout the study. The cynomolgus macaques used in this study were found to be filovirus-, STLV-1, SIV-, and Herpes B- antibody-negative in testing prior to initiation of the study. Male and female cynomolgus macaques of >4kg in weight received intramuscular injections containing 3 mg (total protein) of VLPs and 0.1 mg of QS-21 adjuvant (kindly provided by Agneus, Lexington, MA). Blood samples were obtained under anesthesia from the femoral vein of monkeys. NHPs were challenged with EBOV (target dose of ≈1000 plaque-forming units (pfu)), SUDV (actual dose of 1100 pfu), or TAFV (actual dose of 5375 pfu) via intramuscular injection. Viremia was assayed by conventional plaque assay [28]. Hematological and blood chemistries (kidney/liver-associated enzymes) were measured as previously described [21, 29]. For blood sampling, nonhuman primates were anesthetized with either Ketamine/Acepromazine at a dosage of 0.1 ml/kg body weight or Telazol at 2–6 mg/kg, intramuscularly, prior to handling and phlebotomy (in accordance with institutional standard operating procedures). After exposure, animals were observed at least twice daily: in the morning by a caretaker and in the afternoon by qualified scientific or veterinary staff. No fewer than four hours will elapse between observations. This ensures that moribund animals will be identified quickly and promptly treated for symptoms or euthanized when necessary. All procedures for animal care and housing are in accordance with the NRC Guide for the Care and Use of Laboratory Animals (1996) and the Animal Welfare Act as amended and standards incorporated in 9 CFR Part 3, 1991. At the onset of clinical signs, as determined by Veterinary staff and study director, animals received an intramuscular injection (IAW SOP AC-09–10) of buprenorphine (Buprenex) at a dosage of 0.01 mg/kg body weight, BID, to provide analgesia during the course of infection. Buprenorphine, when given, was administered no less than twice daily during the light phase of the light cycle. The ‘onset of clinical signs’ will be determined by adding the clinical score of the animal as calculated using an IACUC approved score sheet. Some of these criteria include recumbancy, anorexia, inappropriate responses to external stimuli, abnormal bleeding, or forced abdominal respiration or dyspnea. Nonhuman primates were euthanized by methods consistent with recommendations of the Panel on Euthanasia of the American Veterinary Medical Association, specifically using barbiturate overdose via intravenous injection. Animals displaying signs of pain and distress following virus infection were euthanized and considered as non-survivors to the lethal challenge based on the standardized scoring system described.

### Humoral immune responses after vaccination

Levels of EBOV-specific antibodies were determined by an ELISA using purified proteins or live virus. Purified GP proteins (GPdTM, lacking the transmembrane region; constructs kindly provided by Dr. Erica Saphire, Scripps Institute) were expressed in 293T cells and harvested from the supernatants before purification using a two-step column chromatography method. VP40 was produced in *E*. *coli* and purified in a similar manner as previously published [12]. For ELISA using purified protein, the antibodies in unknown samples were determined based on a standard curve of a positive control sera derived from hyperimmune or convalescent animals that was detected by anti-human IgG-HRP. The positive control sera, also referred to as the Reference Detection Antibody (RDA), have been well-characterized using 4-parameter (4PL) curve fit. The value (dilution) at the 4PL inflection point (50% maximum response) was used to establish the number of Antibody Units for the RDA. The RDA with an assigned value of Antibody Units was tested as a reference standard curve on every ELISA plate, to quantify the Antibody Units of the unknown (test) samples. To perform an ELISA using live virus (EBOV, SUDV, TAFV, and Lassa virus) as the coating antigen, plates were coated with a target of 10,000 pfu/well. Serum samples were serially-diluted (semi-log) and detected using an anti-human IgG-HRP followed by addition of TMB substrate. The data are expressed as the means of the endpoint dilution (endpoint defined as inverse of last dilution with an OD > 0.2).

### Statistical analysis

Antibody responses were compared using a student’s t-test or repeated measures ANOVA (mixed model) analysis model as indicated. To reduce the number of monkeys used, only one or two animals per group will be used as a control for uniformly lethal viruses EBOV and SUDV; therefore, use of more control monkeys cannot be justified and historical control monkeys are utilized for data analysis.

## Results

### Two vaccinations provide protection against Ebola virus infection

Our previously published studies demonstrated the ability of a three-dose regimen of EBOV (expressed in 293T cells) [21] or MARV [expressed in insect cells using baculovirus] VLPs containing GP, VP40 and NP [22] to protect cynomolgus macaques from subsequent infection with lethal doses of EBOV or MARV. To determine whether fewer doses of vaccine could confer protection, we conducted a study in which macaques were vaccinated with one or two doses of an EBOV VLP vaccine produced in Hi5 insect cells using a baculovirus expression system. As previously discussed [11, 30], this production strategy was selected due to ease and scale of future manufacturing as well as regulatory experience with the production platform. One or two doses (6 weeks apart) of EBOV VLPs were co-administered with QS-21 adjuvant and vaccinations were timed so that all the macaques were challenged with a target dose of 1000 pfu of EBOV 4 weeks after the final vaccine dose. The antibody responses were determined by ELISA using purified GP (with truncated transmembrane domain, denoted GPdTM) or purified VP40 protein. Macaques vaccinated with 2 doses developed significantly higher antibody responses to GP and VP40 when compared to those receiving only a single dose (**Table 1**, p = 0.0014 and p = 0.0029, respectively using a 2-tailed t-test). After a single EBOV VLP vaccination 2 of 3 vaccinated monkeys were protected from lethal EBOV infection and following 2 vaccinations, all three vaccinated monkeys were protected from EBOV, unlike the single naïve control animal that succumbed to infection on day 7 post lethal EBOV challenge (**Table 1**). The disease observed in the macaques that did not survive the lethal EBOV challenge was consistent with other studies presented in our and previous publications [21, 31, 32]. This study suggested that, using VLPs expressing the three EBOV proteins, immunity can occur within 1–2 vaccinations. Therefore, subsequent vaccination studies in macaques used the two-dose vaccine schedule.

**Table 1:** Protection afforded by eVLP vaccines after 1 or 2 doses in cynomolgus macaques.

		Antibody Units (EC_50_)		Outcome of Challenge with EBOV
Animal	Vaccine	EBOV GPdTM	EBOV VP40	
EVLP1	1 dose EBOV VLP +	93	1379	Survived
EVLP2	QS-21	19	3244	Died, day 6 post challenge
EVLP3		117	3082	Survived
EVLP4	2 doses EBOV VLP +	387	7373	Survived
EVLP5	QS-21	320	9077	Survived
EVLP6		358	7337	Survived
Control	None	2	2	Died, day 7 post challenge

Macaques were vaccinated with 3 mg of EBOV VLPs (total protein content) in 100ug of QS-21 at 6 week intervals with all challenges of ∼1000 pfu EBOV occurring 4 weeks after the final vaccination. Antibody responses were determined using an ELISA to detect IgGs against purified EBOV GPdTM or VP40 in sera drawn immediately prior to challenge.

### VLPs protect against Sudan virus

**Fig 1:**
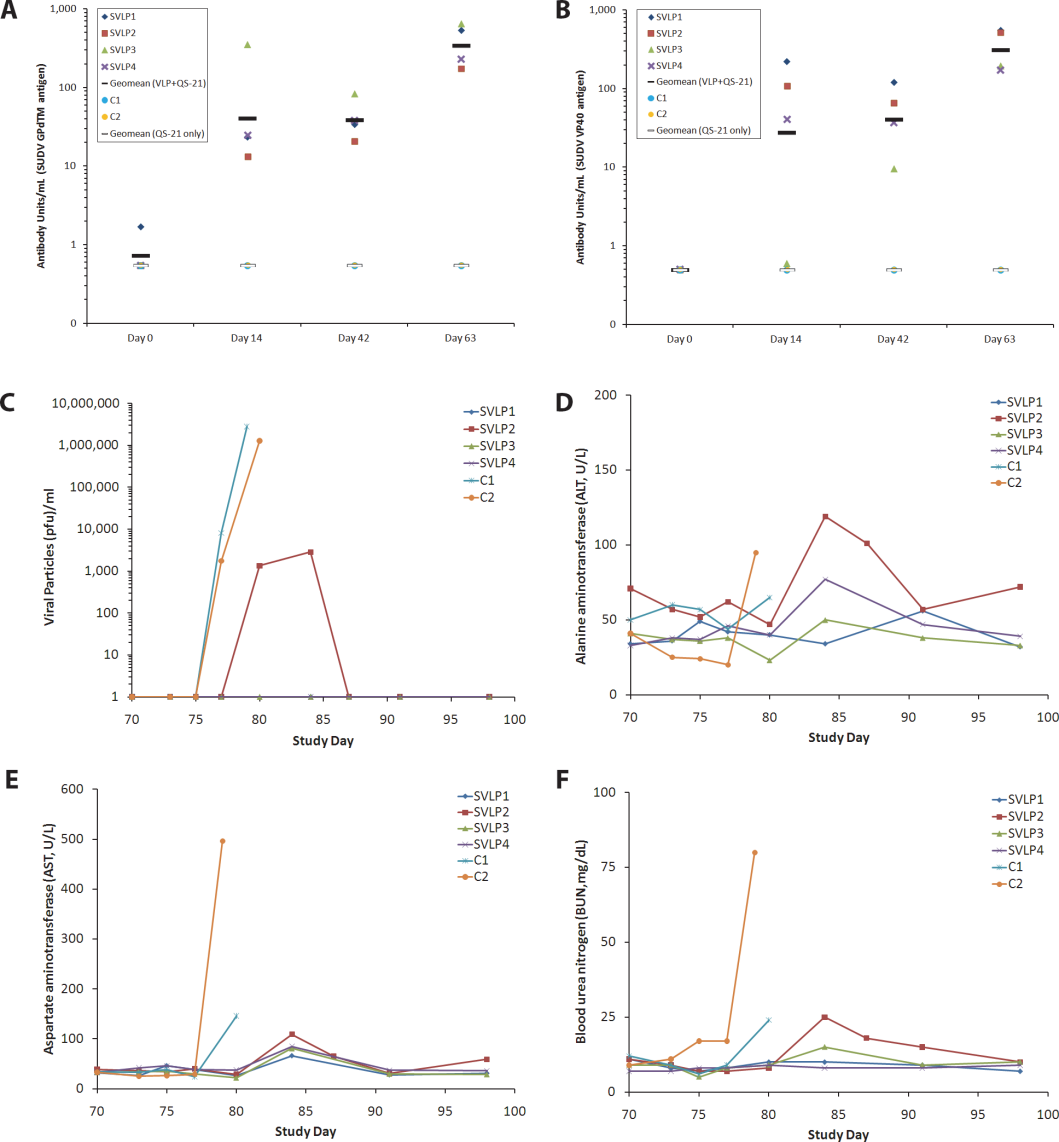
Serology responses following SUDV VLP + QS-21 (SVLP) or QS-21 only (Control, C) vaccination and clinical laboratory analysis following virus challenge. (A-B) Antibody titers from vaccinated animals were measured using the purified SUDV GPdTM (A) or VP40 (B) IgG ELISA. The data are expressed as the antibody units for each animal at each time point and the geometric mean for each group is shown by the lines, as indicated in the figure legends. (C) Infectious virus detectable in the sera of NHPs challenged with SUDV on study day 70 was determined using a standard plaque assay; data are represented as the values for individual monkeys. (D-F) Serum samples were collected at multiple time points during the challenge portion of the study and assayed for clinical chemistry/enzymatic levels including (D) alanine aminotransferase, (E) aspartate aminotransferase, and (F) blood urea nitrogen.

Previous studies showed that animals otherwise protected against EBOV succumbed upon SUDV infection [33] suggesting that EBOV VLPs would not be able to protect against SUDV. Thus, in this study, we sought to determine whether a SUDV VLP vaccine could protect cynomolgus macaques against lethal infection with SUDV. VLPs expressing the SUDV GP, NP, and VP40 were generated in Sf9 cells and co-administered with QS-21 adjuvant to a group of cynomolgus macaques on study days 0 and 42. The four SUDV VLP+QS-21 vaccinated macaques developed high IgG antibody titers against the SUDV GPdTM and VP40 proteins, as assessed by ELISA (**Table 2** and **Fig. 1A-B**). In contrast, two negative control animals, which were vaccinated only with QS-21 adjuvant, did not demonstrate antibody titers to the SUDV proteins (**Table 2** and **Fig. 1A-B**). For the SUDV VLP vaccinated animals (SVLP1–4), the antibody response significantly increased over time compared to placebo (QS-21, p<0.001). On study day 70, the six study animals were challenged intramuscularly with SUDV (Boniface isolate, actual challenge dose of 1100 pfu). Both control animals succumbed with Ebola hemorrhagic fever-like symptoms including fever, maculopapular/cutaneous rashes, anorexia, mild dehydration, and depression. Control animals had detectable viremia by day 77 with a further increase to levels reaching 2x10^6^ PFU/ml at the time of euthanasia on day 79 and day 80 as determined by plaque assay (**Fig. 1C**). Transient, low level of viremia (<2x10^3^ PFU/ml) was observed in one vaccinated NHP (SVLP2) between days 9 and 14 post challenge (**Fig. 1C**). Using quantitative real time polymerase chain reaction (QRTPCR), more than 10^7^/ml genome copy numbers were detected in control animals, while SVLP2 showed a peak copy number of 3.8x10^4^ on day 85 (15 days post challenge). Genome copies were detected also at a low level (<500 copies/ml) in two of the animals (SVLP1 and SVLP3), while one animal (SVLP4) showed no circulating viral genome copies. Clinical chemistry levels (ex. AST, ALP, ALT, BUN, and Cre) increased in the QS-21 adjuvant only control animals (**Fig. 1D-F** and data not shown). Of the vaccinated animals only SVLP2 showed a moderate and transient elevation of ALT, AST, and BUN (**Fig. 1D-F**). During the course of the SUDV infection, the total lymphocyte and platelet counts were significantly decreased in the control animals reaching 50–25% of the baseline values before euthanasia (data not shown). Consistent with the observed transient viremia and clinical chemistry findings, SVLP2 also showed decreased lymphocyte counts on days 10–15 post challenge. Platelet counts were not significantly decreased in any of the vaccinated animals.

**Table 2:** SUDV VLP vaccine protects cynomolgus macaques from lethal infection with SUDV (Boniface isolate).

Animal ID	Vaccine	Antibody Units (EC_50_)		Outcome of Challenge with SUDV
		SUDV GPdTM	SUDV VP40	
SVLP1		540	591	Survived
SVLP2	SUDV VLPs	230	180	Survived
SVLP3	+ QS-21	653	206	Survived
SVLP4		175	550	Survived
Control1	QS-21 only	0.5	0.5	Died, day 10 post challenge
Control 2		0.5	0.5	Died, day 9 post challenge

Macaques were vaccinated with 3 mg of SUDV VLPs (total protein content) along with 100ug of QS-21 at 0 and 6 weeks. Antibody responses were determined using an ELISA to detect IgGs against purified SUDV GPdTM or VP40 in sera drawn immediately prior to challenge. All six macaques were challenged with ∼1000 pfu of SUDV 4 weeks after the final vaccination (week 10).

Both control animals met euthanasia criteria with Ebola hemorrhagic fever-like symptoms on study days 79 and 80 (days 9 and 10 post-challenge, respectively, see **Table 2**). All four VLP-vaccinated macaques survived the SUDV challenge and lived to study completion (study day 98 or day 28 post-challenge). Three of the four SUDV VLP-vaccinated animals (SVLP1, SVLP3, SVLP4) showed minimal symptoms of disease including one or more of the following signs: slight fever, challenge site injection reaction, and/or mild serum chemistry or hematology changes noted between study days 80 and 84 (**Fig. 1D-F**). One vaccinated animal (SVLP2) had moderate signs of disease consistent with viremia, elevated ALT, AST, and BUN, as well as decreased lymphocyte counts as described above. The overall conclusion from these studies is that the VLP expressing SUDV proteins co-administered with QS-21 adjuvant can protect cynomolgus macaques from lethal infection with SUDV; however, vaccination does not lead to sterile immunity as clearly evidenced by detectable virus genomes after challenge.

### Cross-protection against TAFV Infection

**Fig 2:**
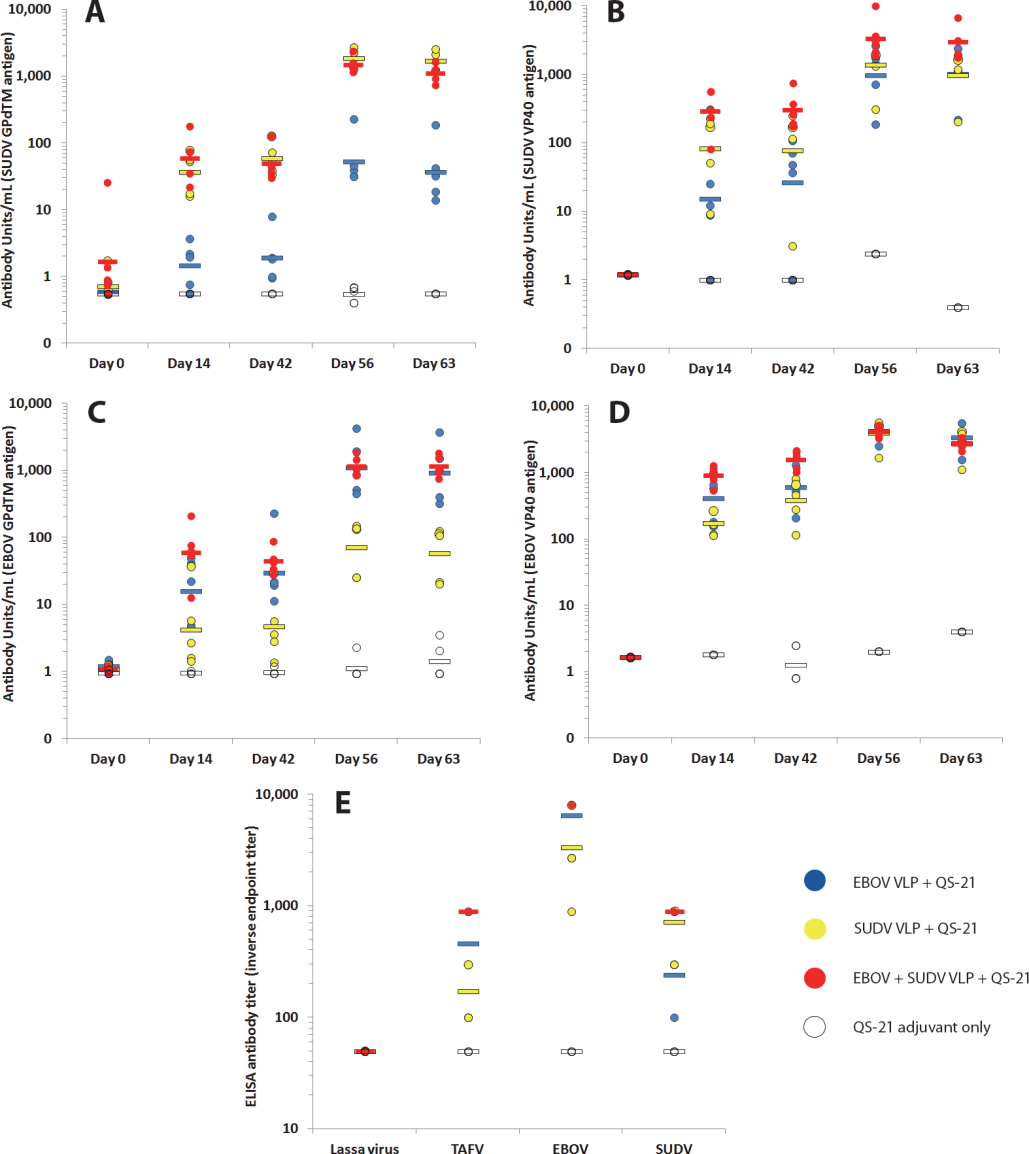
Humoral responses of VLP-vaccinated macaques. The data are shown as individual animal responses (circles) or as the mean antibody units for each group (lines) at each time point the samples were drawn. (A-D) Serum titers of VLP-vaccinated macaques were measured using the purified (A) SUDV GPdTM, (B) SUDV VP40, (C) EBOV GPdTM or (D) EBOV VP40 proteins using an IgG detection ELISA. (E) Antibody response to live virus in VLP-vaccinated animals prior to challenge. An ELISA was performed using live virus (EBOV, SUDV, TAFV, and Lassa virus) as the coating antigen. The data in Panels A-D represent empirically defined EC_50_ values as described in Materials and Methods and data in Panel E are expressed as the means of the endpoint dilution (endpoint defined as inverse of last dilution with an OD > 0.2).

Phylogenetically, TAFV is situated between EBOV and SUDV with closer overall relationship to EBOV than SUDV. At the amino acid level the spike glycoprotein, believed to be the primary protective antigen, shows 56% sequence identity between SUDV and TAFV and 64% sequence identity between EBOV and TAFV. Therefore, we hypothesized that a vaccine based on EBOV or SUDV sequences, or a combination thereof, would provide heterologous protection against infection with TAFV. To this end, cohorts of five macaques were vaccinated with Sf9 cell-produced EBOV VLPs (Group 1), SUDV VLPs (Group 2), a combination of SUDV and EBOV VLPs (Group 3) along with QS-21 adjuvant, or QS-21 adjuvant only (Group 4) at 6-week intervals (study days 0 and 42). The SUDV VLP + QS-21 vaccinated macaques developed high IgG antibody titers against the SUDV GPdTM and VP40 proteins, as tested by ELISA (**Fig. 2A-B**). Similarly, the EBOV VLP + QS-21 vaccinated macaques developed high IgG antibody titers against the EBOV proteins (**Fig. 2C-D**). The macaques vaccinated with a combination of SUDV and EBOV VLPs demonstrated high antibody titers to all four (SUDV and EBOV GPdTM and VP40) proteins, which were similar to homologous responses in the groups where only one VLP type was administered (**Fig. 2A-D**). Heterologous antibody responses (eg. SUDV VLP vaccinated macaques against EBOV proteins and vice versa) were also observed but were slightly lower (∼1 log) than the homologous responses (p<0.005 for all comparisons using mixed model ANOVA). In contrast, the cynomolgus macaques vaccinated with QS-21 adjuvant alone did not demonstrate appreciable antibody titers (above background threshold in ELISA) to the SUDV or EBOV recombinant proteins (**Fig. 2A-D**) indicating that the responses are specific. Since purified TAFV GP protein was not available to directly measure the heterologous responses to this ebolavirus, the serum antibody titers against live TAFV as well as SUDV, EBOV, or Lassa virus (as a negative control) were also determined for each animal on study day 63. All VLP-vaccinated macaques demonstrated reactivity to the three live EBOV antigens tested (**Fig. 2E**). Those vaccinated with EBOV or EBOV+SUDV VLPs (groups 1 and 3) had the highest mean antibody titers against TAFV. As expected, heterologous responses were also evident between EBOV and SUDV and the homologous responses were further enhanced by the combined vaccination (**Fig. 2E**). The macaques in group 4 (QS-21 only) did not show appreciable titers to the EBOV antigens, and none of the macaques in the study demonstrated reactivity against the negative control Lassa virus (**Fig. 2E**).

**Fig 3:**
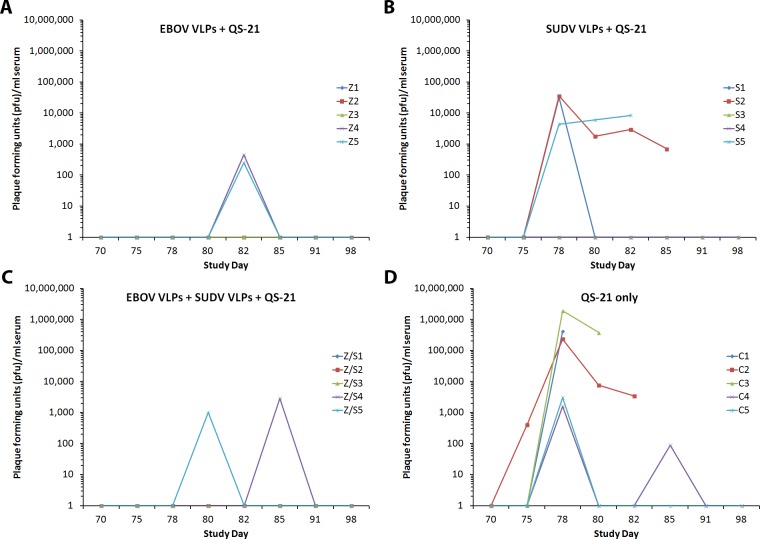
Infectious virus detectable in the sera of NHPs vaccinated with QS-21 adjuvant and EBOV VLPs (A), SUDV VLPs (B), EBOV and SUDV VLPs (C), or adjuvant only (D) and challenged with TAFV on study day 70. Quantitation of virus infectivity was determined using a standard plaque assay and is represented as the values for individual monkeys on each study day after the challenge date (day 70).

All macaques were challenged via intramuscular injection with 5375 pfu of live TAFV on study day 70 (4 weeks after the second vaccination). Three of the five (60%) control animals vaccinated with QS-21 alone succumbed with classic filovirus symptoms and pathology on study days 80–82 (i.e., days 10, 10, and 12 post-challenge, respectively), while the remaining two control animals developed severe disease symptoms but recovered (**Table 3**), consistent with previous report showing 60% lethality by TAFV [34]. All five macaques vaccinated with EBOV VLPs and QS-21 adjuvant were protected from TAFV infection, with only a single macaque exhibiting minor clinical changes including fever and decreased platelet count on study days 78–80 and two animals showing transient low viremia on study day 82 (**Table 3**). In contrast, only three of five macaques vaccinated with SUDV VLP survived, with one exhibiting notable clinical changes on study days 75–82 and the remaining two macaques exhibiting moderate symptoms of infection including mild rash and fever (**Table 3**). Concurrent administration of EBOV and SUDV VLPs with QS-21 adjuvant protected all five macaques from lethal infection, with a single animal exhibiting significant clinical signs including fever, anorexia, and weight loss, and other animals showing minor and transient symptoms (**Table 3**). The level of infectious virus in blood was determined for each TAFV-infected macaque, and viremia mirrored the clinical symptoms observed in the animals. Animals succumbing to disease had the highest viral titers, while those being protected from hemorrhagic fever exhibited detectable viremia (**Fig. 3**). Increased WBC counts were observed in four out of five control animals. Transient increases in WBC were also seen in some of the surviving vaccinated animals; however, these changes did not correlate with clinical outcome. In contrast most symptomatic animals consistently showed decreased platelet and lymphocyte counts as well as elevated liver enzymes and BUN (**Table 3**). The overall conclusion from this study is that vaccination of cynomolgus macaques with the EBOV VLPs or a mixture of EBOV and SUDV (both with QS-21 adjuvant) can provide protection against a TAFV infection.

**Table 3:** Summary of the TAFV challenge study results through 15 days post challenge (study day 70–85).

	NHP #	d70–75	d78	d80	d82	d85	Outcome
	Z1	N.S.	Fever, ↓Plt, ↓ALB	↓Plt, ↓ALB	N.S., ↓ALB	N.S.	Survived
**Group 1**	Z2	N.S.	N.S.	N.S.	N.S.	N.S.	Survived
**EBOV**	Z3	N.S.	N.S.	N.S.	N.S., viremia	N.S.	Survived
**VLP**	Z4	N.S.	N.S.	N.S.	N.S.	N.S.	Survived
	Z5	N.S.	N.S.	WL 5–10%	WL 5–10%, viremia	WL 5–10%	Survived
	S1	Fever	Fever, rash, ↑AST, ↓Lym	Fever, rash, ↓Plt, ↑BUN, ↑ALT, ↑↑↑AST, WL 5–10%	↓Plt, ↑ALT, ↑AST, ↑BUN, ↑Glu, ↓TP, WL 5–10%	↓TP, ↓ALB, ↑WBC, WL 5–10%	Survived
**Group 2**	S2	Fever	Fever, ↓Plt, ↑AST, ↑Cre, viremia	Fever, mildly unresponsive, ↓Plt, ↑ALT, ↑↑AST, viremia	Fever, moderately unresponsive, anorexia, ↑↑↑BUN/ALT/AST, viremia	Temp. drop, WL15%, labored breathing, dehydration, ↑↑↑BUN/ALT/AST, viremia	Euthanized day 15
**SUDV**	S3	N.S.	mild rash	mild rash	N.S.	N.S.	Survived
**VLP**	S4	N.S.	Fever	mild rash	N.S.	N.S.	Survived
	S5	↓Plt,	Fever, mild rash, ↓Plt,	WL 5–10%, mild rash, ↓Plt,↑↑BUN, ↑AST, viremia	WL 15%, dehydration, moderately unresponsive, anorexia, mild rash, ↑↑AST, viremia	N/A.	Euthanized day 12
	Z/S1	Fever	N.S.	N.S.	N.S.	N.S.	Survived
**Group 3**	Z/S2	N.S.	Fever	WL 5–10%	WL 5–10%, dehydration	WL 5–10%	Survived
**EBOV+**	Z/S3	N.S.	↑WBC	Dehydration	N.S.	N.S.	Survived
**SUDV**	Z/S4	N.S.	↑WBC	N.S.	N.S.	N.S. viremia	Survived
**VLP**	Z/S5	N.S.	N.S.	N.S., viremia	N.S.	N.S.	Survived
	C1	↑ALT	Fever, rash, anorexia, ↓Plt, ↑BUN, ↑↑↑ALT/AST, viremia	N/A	N/A	N/A	Found dead day 10
**Group 4**	C2	N.S.	Fever, ↓Plt, ↑↑AST, viremia	Fever, mildly unresponsive,mild rash, ↓Plt, ↑BUN, ↑↑↑ALT/AST, ↑↑ALP, viremia	Fever, WL 5–10%, rash, anorexia, ↓Plt, ↑↑↑BUN/ALT/AST, ↑ALP, viremia	N/A	Euthanized day 12
**QS-21**	C3	N.S.	↓Plt, ↑ALT, ↑↑↑AST, viremia	Temp. drop, labored breathing, rash, anorexia, ↑WBC, ↓Plt, ↑↑↑BUN/ALT/AST, ↑ALP, viremia	N/A	N/A	Euthanized day 10
	C4	N.S.	↑BUN, ↑AST, viremia	WL 5–10%, edema, ↑BUN, ↑↑↑ALT/↑AST	WL 5–10%, ↑BUN, ↑↑↑ALT/↑AST	WL 5–10%, ↑ALT, viremia	Survived
	C5	N.S.	Fever, ↑↑↑AST, viremia	↑BUN, ↑ALT, ↑↑↑AST	↑BUN, ↑ALT, ↑AST	↑ALT	Survived

*N*.*S*. = *no signs; N/A = not determined because the animal was deceased prior to the time point; fever was defined as a temperature more than 2*.*0°F over baseline; Temp drop was defined as a temperature more than 3*.*0°F below baseline; mild rash = focal areas of petechiae covering less than 10% of skin; moderate rash = areas of petechiae covering between 10% and 40% of the skin; severe rash = areas of petechiae or ecchymosis covering more than 40% of the skin*

*↑*, *2- to 3-fold increase*

*↑↑*, *4- to 5-fold increase*

*↑↑↑*, *> 5-fold increase*

*↓*, *2- to 3-fold decrease*

*WL*: *weight loss was the percentage compared to the weight at study initiation; BUN*: *blood urea nitrogen; ALT*: *alanine aminotransferase; AST*: *aspartate aminotransferase; ALP*: *alkaline phosphatase; ALB*: *albumin*, *Glu*: *glucose*, *Cre*: *creatinine*, *WBC*: *white blood cells; Plt*: *platelet*, *Lym*: *lymphocyte*.

## Discussion

The current studies extend our previous data in nonhuman primates demonstrating that the filovirus VLP vaccines are a promising candidate for protection against lethal infection. Filovirus VLPs can be produced in mammalian or insect cell expression systems. Our previous studies demonstrated the efficacy of the mammalian produced VLPs against EBOV in rodents [8, 35] and macaques [21]. We have also demonstrated the efficacy of insect cell produced VLPs against EBOV in rodents [11, 35] and against MARV in NHPs [22]. In this study, we demonstrate complete protection against homologous lethal infection by two doses of baculovirus/insect cell-derived EBOV VLPs and partially protective effects after a single vaccination. The dose, as measured by total protein, of baculovirus/insect cell-derived EBOV VLPs used in the current studies was higher than the previously published study using mammalian VLPs in macaques but similar to that used to vaccinate chimpanzees [36]. Future studies will address the minimum effective dose and regimen for the baculovirus/insect cell-derived EBOV VLPs; however, previous studies in mice suggest that baculovirus/insect cell-derived VLPs are more potent that mammalian-derived VLPs [11]. Additionally, as described in the current report, SUDV VLPs were generated and shown to be efficacious against a lethal homologous virus infection. Along with a previous reports on the efficacy of alphavirus-based replicon [37] and VSV-based [34] vaccines, this is the third report of an efficacious vaccine strategy in nonhuman primates against Sudan virus. Moreover, for the first time, we have demonstrated the ability of an EBOV-based vaccine to provide complete protection in nonhuman primates against the heterologous ebolavirus, TAFV. Previous vaccine studies with virus-vectored platforms had used a combination of SUDV and EBOV antigens to protect against TAFV or BDBV [34, 38], and a recent study showed partial protection against BDBV following administration of a VSV-vectored EBOV vaccine [26].

While both T and B cell responses are reported to play a role in protective immune responses to filoviruses, a series of recent reports indicate that antibody alone can provide significant protection. Dye *et*. *al* showed that purified convalescent IgG from macaques can protect NHPs against infection with MARV and EBOV when administered as late as 48h post exposure [39]. Olinger *et*. *al* reported significant protection from EBOV infection in NHPs treated with a cocktail of three monoclonal antibodies to GP administered 24h and 48h post exposure [40]. Similar results were also reported in two other studies combining monoclonal antibodies with adenovirus-vectored interferon alpha therapy in guinea pigs [41] and NHPs [42]. Collectively these data suggest a key role for the humoral response in controlling filovirus infection. Based on these findings immunological analyses in this current vaccine study were focused on the humoral antibody responses.

Previous kinetic studies of the antibody titers and T cell responses in the EBOV and MARV VLP-vaccinated nonhuman primates seemed to indicate that the third dose of VLPs did not drastically boost the humoral response, as compared to those observed upon two vaccinations [21, 22]. In the current studies, macaques vaccinated with a single dose of EBOV VLPs developed lower antibody responses to the protective glycoprotein antigen than the macaques vaccinated with two doses. The macaque from the single vaccine dose group having the lowest antibody response to GP succumbed to the lethal EBOV challenge. This animal indeed had a high VP40 titer similar to the other two animals further suggesting the importance of anti-GP antibodies for protection. The remaining two single dose VLP-vaccinated monkeys were protected from lethal EBOV infection (**Table 1**). Following two vaccinations, all macaques showed increased anti-GP antibody titers and were protected from EBOV infection. Based on these data all consequent studies were performed using the shorter vaccination regimen.

Previous studies had not addressed the protective potential of filovirus VLPs against SUDV infection. In the current study we determined whether the SUDV VLPs could protect macaques upon administration of two doses of VLPs comprised of the homologous GP, NP and VP40 that was co-administered with QS-21 adjuvant. Our results show that SUDV VLPs are able to induce high IgG responses and provide full protection from lethal SUDV infection. Vaccine-induced immunity was not uniformly sterile, as one of the vaccinated macaques (SVLP2) showed transient viremia and moderate symptoms that included weight loss and injection site reactions, as well as blood chemistry changes. The remaining three animals in the vaccine group had minor clinical signs of infection but controlled the infection well in comparison to SUDV-infected naïve animals from other historical vaccine studies. Despite the transient symptoms, all vaccinated animals recovered by no later than 20 days post infection. The overall conclusion from these studies is that the VLP vaccine expressing SUDV viral proteins can induce IgG antibodies recognizing SUDV GP and VP40 in VLP-vaccinated macaques, and that vaccination can protect cynomolgus macaques from infection with the lethal isolate SUDV-Boniface. It is possible that a third dose of vaccine might confer complete protection against both lethality and viremia, as has been seen for both EBOV and MARV [21, 22].

The purpose of the third study was to examine the immunologic and protective activity of EBOV and SUDV VLPs (independently or in combination) against a heterologous infection with TAFV. All VLP-vaccinated macaques developed high IgG antibody titers against both EBOV and SUDV GPdTM, and VP40 proteins as tested by ELISA. As expected, the vaccination induced higher antibody titers against homologous antigens compared to heterologous responses. Importantly, vaccination with the blended mixture of EBOV and SUDV VLPs did not appear to induce immunologic interference but rather slightly amplified responses to both the SUDV and EBOV antigens, especially at earlier time points during the course of vaccination. The survival outcome of our control adjuvant-only cohort (40% survival, three of five animals succumbed to challenge) is in agreement with the published historical data from studies utilizing the same virus stock of TAFV [34]. The results from the challenge portion of the study indicated that vaccination with EBOV VLP plus QS-21 adjuvant, at the given vaccination schedule and dose, provides protection from TAFV infection. In contrast, these data suggested that vaccination with SUDV VLP is not sufficient to protect NHPs from this exposure. This is the first study to examine the ability of EBOV or SUDV vaccines alone to protect against a heterologous infection with TAFV, as other vaccine studies used both EBOV and SUDV GP components [34, 38]. The study is also the first demonstration of full cross-species protection by an EBOV-based vaccine. A recent study using a vesicular stomatitis virus (VSV) vector expressing EBOV GP provided partial protection against BDBV infection [26]. The drastic difference between the efficacy of EBOV and SUDV VLPs was somewhat surprising, since EBOV and TAFV share only 63% identity based on whole genome sequences while SUDV and TAFV have only slightly lower full genome sequence identity at ∼58% [1]. Given the small number of animals in each group and the complication that TAFV is not 100% lethal in animal models, more studies are needed to confirm the observation that SUDV VLPs do not protect against TAFV.

In the future, it will be necessary to demonstrate the ability of the VLP vaccines to protect against BDBV having whole genome sequence identities of 63% to EBOV, 58% to SUDV and 68% to TAFV [1]. Since both the TAFV and BDBV models of infection in cynomolgus macaques are only partially lethal (60% and 75%, respectively), future studies with these models require larger numbers of animals [26, 34, 38]. Nonetheless, these findings clearly suggest that a broadly reactive panfilovirus VLP vaccine formulation could be feasible.

## Supporting Information

S1 Arrive Checklist ARRIVE checklist. (PDF)
